# The Bacteriostatic Activity of 2-Phenylethanol Derivatives Correlates with Membrane Binding Affinity

**DOI:** 10.3390/membranes11040254

**Published:** 2021-03-31

**Authors:** Isabel S. Kleinwächter, Stefanie Pannwitt, Alessia Centi, Nadja Hellmann, Eckhard Thines, Tristan Bereau, Dirk Schneider

**Affiliations:** 1 Department of Chemistry, Biochemistry, Johannes Gutenberg University Mainz, Hanns-Dieter-Hüsch-Weg 17, 55128 Mainz, Germany; kisabels@uni-mainz.de (I.S.K.); Stefanie.Pannwitt@gmx.de (S.P.); nhellmann@uni-mainz.de (N.H.); 2 Max Planck Institute for Polymer Research, 55128 Mainz, Germany; centia@mpip-mainz.mpg.de (A.C.); t.bereau@uva.nl (T.B.); 3 Institute of Molecular Physiology, Johannes Gutenberg University, Hanns-Dieter-Hüsch-Weg 17, 55128 Mainz, Germany; thines@uni-mainz.de; 4 Van ‘t Hoff Institute for Molecular Sciences and Informatics Institute, University of Amsterdam, 1098 XH Amsterdam, The Netherlands

**Keywords:** 2-phenylethanol, phenylacetic acid, phenyllactic acid, methyl phenylacetate, Tyrosol, biomembranes, membrane interaction, bacteriotoxic

## Abstract

The hydrophobic tails of aliphatic primary alcohols do insert into the hydrophobic core of a lipid bilayer. Thereby, they disrupt hydrophobic interactions between the lipid molecules, resulting in a decreased lipid order, i.e., an increased membrane fluidity. While aromatic alcohols, such as 2-phenylethanol, also insert into lipid bilayers and disturb the membrane organization, the impact of aromatic alcohols on the structure of biological membranes, as well as the potential physiological implication of membrane incorporation has only been studied to a limited extent. Although diverse targets are discussed to be causing the bacteriostatic and bactericidal activity of 2-phenylethanol, it is clear that 2-phenylethanol severely affects the structure of biomembranes, which has been linked to its bacteriostatic activity. Yet, in fungi some 2-phenylethanol derivatives are also produced, some of which appear to also have bacteriostatic activities. We showed that the 2-phenylethanol derivatives phenylacetic acid, phenyllactic acid, and methyl phenylacetate, but not Tyrosol, were fully incorporated into model membranes and affected the membrane organization. Furthermore, we observed that the propensity of the herein-analyzed molecules to partition into biomembranes positively correlated with their respective bacteriostatic activity, which clearly linked the bacteriotoxic activity of the substances to biomembranes.

## 1. Introduction

Due to their amphipathic properties, alcohols affect numerous biological processes, many of which are related to cellular membranes. The hydrophobic tails of alcohols insert into the hydrophobic core region of a lipid bilayer and disrupt hydrophobic interactions between the lipid molecules, resulting in a decreased lipid order, i.e., an increased membrane fluidity [1,2]. The exact impact of alcohol on the structure of a lipid bilayer depends on the length and overall hydrophobicity of the alcohol alkyl chain, and it is predicted that the effect of alcohols on membranes increases with increasing alkyl chain lengths [3]. Thus far, the impact of alcohols on biomembranes has been studied to a great extent using, aliphatic primary alcohols, albeit aromatic alcohols, such as 2-phenylethanol (2-PEtOH), also insert into lipid bilayers and disturb the membrane organization [4].

2-PEtOH, a compound also known as phenylethyl alcohol or benzylcarbiol, is a colorless liquid with a rose-like odor. 2-PEtOH occurs widely in nature and is—besides in rose extracts—a major component in a variety of plant extracts from carnations, hyacinths, jasminum, geranium species, and others [5]. 2-PEtOH has been shown to affect cell proliferation in bacteria, yeast, plants, fungi, and mammalian cells, albeit its exact mode of action is still under debate [6,7,8,9]. Due to its bactericidal effect, 2-PEtOH is frequently used in concentrations of up to 100 mM to protect pharmaceuticals, cosmetics, and other personal care products from spoilage. However, 2-PEtOH is already bacteriostatic at vastly lower concentrations, starting at concentrations as low as 8 mM with explicit effects at 12–16 mM [7]. 

2-PEtOH appears to affect DNA, RNA, and protein synthesis in bacteria [10,11,12]. Additionally, it has been suggested that the bactericidal activity of 2-PEtOH in fact depends on its conversion into phenylacetaldehyde, which is way more toxic to bacteria than 2-PEtOH [12]. Yet, while diverse targets are discussed to be causing the bacteriostatic activity of 2-PEtOH, it is clear that 2-PEtOH partitions into bacterial membranes and severely affects the structure of biomembranes [4,7,13]. As observed with other alcohols, the interaction of 2-PEtOH with the model as well as with biological membranes results in a drastic change of the lipid acyl-chain order [14,15,16]. This 2-PEtOH-induced change in the lipid order significantly affects the dimerization of transmembrane helices in the model, as well as in cellular membranes [14]. Thus, 2-PEtOH-induced lipid disordering might crucially affect the structure of transmembrane proteins in general, and this effect on the structure of biological membranes might be the first and ultimate line of the 2-PEtOH bacteriostatic activity. 

**Figure 1:**
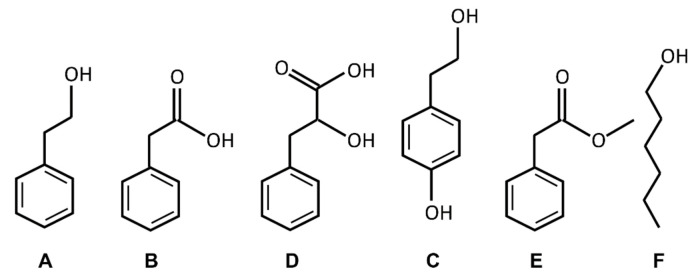
Chemical structures of 2-phenylethanol (2-PEtOH) and derivatives. 2-phenylethanol (**A**), phenylacetic acid (**B**), phenyllactic acid (**C**), Tyrosol (**D**), methyl phenylacetate (**E**), and 1-hexanol (**F**). The structures were drawn with ChemSketch V5.

Further, 2-PEtOH is also produced by some fungi, which can significantly retard their growth and development [6,9]. Yet, fungi also produce some 2-PEtOH-derivatives, such as Tyrosol, phenyllactic acid, and phenylacetic acid (Figure 1), some of which appear to also have bacteriostatic activities. Already decades ago, a correlation between the bacteriostatic activity of phenyl-substituted alcohols (other than analyzed here) and their partitioning between an aqueous phase and an organic layer was described, and it was suggested that the compounds acted on cellular membranes [13]. Thus, it is reasonable to assume that the 2-PEtOH derivatives also act on cellular membranes and affect cell viability.

Similar to 2-PEtOH, phenyllactic acid, the metabolite of phenylethylamine that occurs in the phenylalanine metabolism [17,18,19], also appears to have antimicrobial properties, targeting fungi and bacteria [14,15,20,21,22]. Just as for 2-PEtOH, also for phenyllactic acid, diverse modes of actions are discussed, and the compound appears to affect the integrity of the bacterial cell wall [14,20], and/or it might intercalate into the DNA and hinder DNA replication [23].

Yet, just as 2-PEtOH, it has been suggested that phenylacetic acid does also act at the cell membrane, albeit not knowing the mode of action [17]. Clearly, phenylacetic acid can passively cross liposomal membranes in vitro [24], and a correlation between the membrane partition coefficients of some of its para-substituents and their bacteriostatic properties is described [25], and phenyllactic acid potentially makes the outer membrane of *Escherichia coli* more permeable, without disrupting it [23]. In contrast to 2-PEtOH and phenylacetic acid, membrane interaction of methyl phenylacetate, the methyl ester of phenylacetic acid, which is produced by several plants, has not been analyzed yet. 

Tyrosol belongs to the most widely distributed compounds in plants [16]. It is the major phenolic compound found in olive oil, red wine and white wine [26,27].

While membrane interaction of 2-PEtOH has been studied to some extent and the bacteriostatic activity of 2-PEtOH has been linked to its membrane activity, the interaction of 2-PEtOH derivatives with model and biomembranes has only marginally been studied, if at all. Given that most compounds have an amphipathic nature, membrane interaction is expected, and it is well possible that membrane interactions affect bacterial homeostasis. In the present study, we showed that the 2-PEtOH derivatives phenylacetic acid, phenyllactic acid, and methyl phenylacetate (Figure 1) were incorporated into the model membranes and affected the membrane structure. The higher the overall hydrophobicity of a 2-PEtOH derivative, the higher its fluidizing impact on a membrane. Furthermore, we observed a positive correlation between membrane partitioning and the bacteriostaticity of the here analyzed 2-PEtOH derivatives.

## 2. Materials and Methods

### 2.1. Lipids and Chemicals 

*E. coli* total lipid extract (EPL) was purchased from Avanti Polar Lipids (Alabaster, AL, USA). Laurdan (6-dodecanoyl-2-dimethylamino) was purchased from Sigma-Aldrich (Munich, Germany).

2-PEtOH, phenylacetic acid, phenyllactic acid, Tyrosol, 1-hexanol, and methyl phenylacetate were all purchased from Sigma-Aldrich (Munich, Germany). All structures were drawn with the program ChemSketch V5 (Freeware from ACD/Labs, Toronto, Ontario, Canada). The partition coefficient (logP value) was calculated using Molinspiration v2016.10 (www.molinspiration.com, accessed on 21 June 2018.).

It can be expected that the effect of a given compound on a membrane correlates with its tendency to incorporate into the membrane, represented by the corresponding partitioning coefficient P. This type of correlation is probed as [28]

(1) $$\log\frac{1}{C}=a\times \mathrm{logP}+b$$

if only logP is considered as the predictor for the activity of the substance, with a and b being constants and C the concentration relevant for the effect to be tested, here: Minimal inhibitory concentration 50 (MIC50; see [28] for a recent discussion of the approach).

### 2.2. Laurdan Fluorescence Spectroscopy and Generalized Polarization (GP) Values

For liposome preparation, 25 µL EPL (10 mM in chloroform and methanol, 2:1) was mixed with laurdan (dissolved in methanol) in a 500:1 molar ratio. The solvents were removed under a nitrogen stream, and the remaining traces of the solvent were removed by overnight vacuum desiccation. The next day, 250 µL of the substances dissolved in 10 mM 4-(2-hydroxyethyl)-1-piperazineethanesulfonic acid (HEPES)-buffer (pH 7.4, 150 mM NaCl) at the desired concentrations were added and the lipid film was rehydrated, resulting in a solution with 1 mM lipid. 

The mixture was vortexed and subsequently incubated for 30 minutes at 37 °C and 1000 rpm on a Thermoblock mixer comfort from Eppendorf. To prepare large unilamellar liposomes (LUVs), five freeze-thaw cycles were performed. The liposomes were again incubated for 30 minutes at 37 °C and 1000 rpm on a Thermoblock mixer comfort from Eppendorf (Hamburg, Germany). Laurdan spectra were recorded at 25 °C using a Fluoro-Max-4 fluorescence spectrometer (Horiba (Bensheim, Germany)). The excitation and emission slits were set to 2 nm, and the spectra were recorded between 400 and 550 nm with excitation at 350 nm. Each tested substance was measured at least three times at each concentration using freshly prepared liposomes. 

The generalized polarization (GP) values were calculated from the fluorescence emission spectra using the following equation [29].

(2) $$\mathrm{GP}=\frac{I_{440}-I_{490}}{I_{440}+I_{490}}$$

I_440_ and I_490_ are the emission intensities at 440 and 490 nm [29]. The range of concentrations employed was limited by the solubility of the tested substances in an aqueous buffer. Tyrosol and 2-phenylethanol were measured up to 100 mM, phenylacetic acid and phenyllactic acid up to 80 mM, 1-hexanol up to 70 mM, and methyl phenylacetate up to 15 mM.

### 2.3. Growth Assay and Determination of Minimal Inhibitory Concentrations 50 (MIC_50_)

*Escherichia coli* strain MC4100 was grown in a terrific broth (TB)-medium buffered with 10 % K_2_HPO_4_/KH_2_PO_4_ (0.17 M/0.72 M). 20 mL TB medium with 1:1000 streptomycin (50 mg/mL) was prepared, and the tested substance was dissolved in the medium at the given concentration. From an overnight culture, *E. coli* MC4100 was diluted in fresh medium to an OD_600_ of 0.2. The cultures were incubated at 37 °C and 200 rpm for four hours, the OD_600_ of a 5fold diluted sample was measured, and the real value was calculated. The highest tested concentration was defined by the solubility of the tested substances in TB-medium or was the concentration where the *E. coli* were not able to grow anymore. A dose-response curve was fit to the data with a modified Hill equation using OriginPro 8.6 (Northampton, MA, USA), [30], with the concentration of the substance x and the parameter e reflecting the MIC50 for each tested substance (Figure 4).

(3) $$y=c+\frac{d-c}{1+{\left(\frac{x}{e}\right)}^{b}}$$

In order to get an idea about the concentration of the substance incorporated into the *E. coli* membrane, we calculated the overall surface area presented by the bacteria and, based on the partition coefficient, calculated the concentration of the lipids required to obtain the corresponding area/ml as liposomes. According to [31], an OD of 0.2 corresponds to about 2 × 10^8^ *E. coli* cells/ml. The surface area of an *E. coli* cell is about 3 µm^2^ [32], corresponding to about 4.2 × 10^6^ lipid molecules, assuming 0.7 nm^2^/lipid [33]. Thus, the concentration of the lipids necessary to create the corresponding area of a lipid bilayer is about 3 µM.

### 2.4. Computer Simulations

We followed previously established simulation protocols that were described in detail elsewhere [34,35]. Coarse-grained simulations were performed using Gromacs 4.6 [36] and the Martini force field [37]. Small molecules were parametrized using the auto-martini scheme [38] and inserted into a symmetric di-linoleoyl-phosphatidyl-choline (DLiPC) bilayer consisting of 64 lipids per leaflet and solvated in water. We ran simulations in the *NPT* ensemble at 300 K and 1 bar. Each simulation included a sequence of minimization, heat-up, and equilibration runs prior to the production one, the latter being simulated for $10^{5}\;\tau \;$ using a time step of δt=0.02 τ, where τ (1 ps) refers to the model’s natural unit of time.

## 3. Results and Discussion

The impact of 2-PEtOH on the structure and the stability of the model, as well as biological membranes, were studied in the past [6,7,8,14,15,16]. Nevertheless, plants, some fungi, as well as some bacteria produce and secrete the 2-PEtOH derivatives phenylacetic acid, phenyllactic acid, and Tyrosol (Figure 1). While it was suggested that phenyllactic acid permeabilizes the outer membrane of bacteria (by a yet unknown mechanism) [23], the membrane activity of the 2-PEtOH derivatives is largely unexplored. All molecules are amphiphilic and have a polar region with hydroxyl or carboxyl groups and a non-polar phenyl ring. An exception is Tyrosol, which has an extra hydroxyl group at the phenyl ring that renders the molecule non-amphiphilic [16]. Furthermore, in the present study, we additionally analyzed methyl phenylacetate, the methyl ester of phenylacetic acid produced in some plants, as it allows separating effects of the polar group from effects potentially caused by the negative charge of the carboxylate group. Furthermore, 1-hexanol, whose membrane interaction is well studied [1,2], was used as a non-aromatic control.

### 3.1. Membrane Partitioning and the Impact of 2-PEtOH Derivatives on the Membrane Structure

To estimate the membrane-binding affinity of the here-analyzed substances, we first calculated logP values (Table 1), which provide information as to the partitioning of the substances between water and octanol (which is typically used as a mimic of the hydrophobic membrane core). This calculation is based on the hydrophobicity and polarity of a substance, and the less hydrophobic a molecule, the lower the logP value [39].

Phenyllactic acid has the lowest logP value and, thus, is the least hydrophobic molecule analyzed here, and 1-hexanol is the most hydrophobic molecule with the highest logP value of our studied substances.

**Figure 2:**
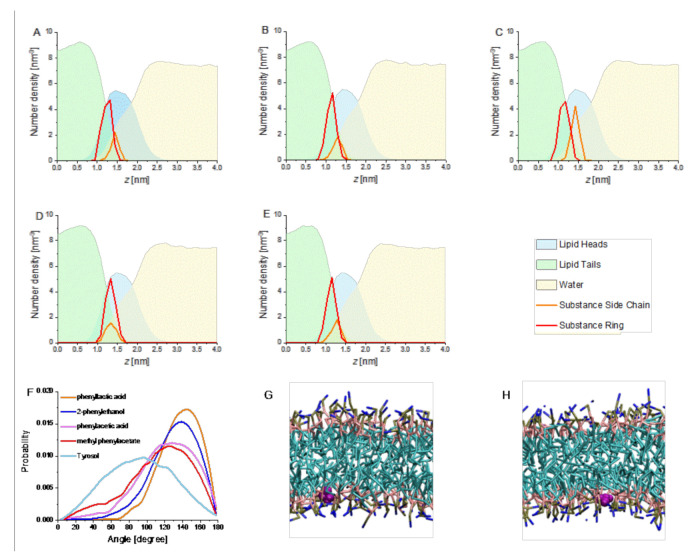
Number–density profiles, compound orientation relative to the membrane normal and simulation snapshots. (**A**–**E**) The number–density profiles report the molar fraction of different chemical groups in the simulation box, perpendicular to the bilayer. (**A**) 2-PEtOH, (**B**) phenylacetic acid, (**C**) phenyllactic acid, (**D**) Tyrosol, and (**E**) methyl phenylacetate. All substances have shifts between the side chain and ring, except Tyrosol. Molar fractions of the solutes have been multiplied by 50 for clarity. All substances spontaneously insert close to the lipid head groups. (**F**) The angle between membrane normal and compound orientation (defined from side chain to aromatic ring) shows large values for methyl phenylacetate, phenylacetic acid, 2-PEtOH, and phenyllactic acid, indicative of their intercalation in the membrane. Smaller values are reported for Tyrosol, indicating its binding parallel to the membrane surface. (**G**,**H**) Representative simulation snapshots of 2-PEtOH and Tyrosol (purple) inserted in the headgroup region of the phospholipid membrane. Water is not shown for clarity.

Based on the definition of the logP, it is evident that a substance with a negative logP has a higher affinity to the aqueous phase, and a positive logP denotes a higher concentration in the lipid phase. Thus, based on this analysis, we expected all our tested substances to incorporate into the membrane (the lipid phase) as all the calculated values are positive [40]. Indeed, computer simulations clearly indicated that all substances incorporated around the lipid head groups (Figure 2). The ring lies deeper in the membrane than the side chain for all substances except Tyrosol. In fact, the angle distribution shown below (Figure 2) indicated that Tyrosol did not intercalate into a membrane but rather bound parallel on a membrane surface, in line with previous assumptions [16,41]. The angle between the membrane bilayer normal and the orientation of Tyrosol was just about 90°, while all other compounds showed much larger angles (Figure 2F). 

Thus, all 2-PEtOH derivatives intercalated into a lipid monolayer, except Tyrosol, where direct interactions with the lipid acyl chains were not expected.

As membrane integration and membrane activity of 2-PEtOH had been demonstrated in the past, we next determined the impact of 2-PEtOH derivatives on the structure of model membranes via laurdan fluorescence spectroscopy [1,2,8,9,14,17,20]. Laurdan is a fluorescent dye that incorporates in lipid bilayers. Changes in the laurdan fluorescence emission spectrum reflect changes in the dye’s ultimate environment, e.g., caused by altered lipid packing. To quantify the impact of a molecule on the structure of a lipid bilayer, the generalized polarization (GP) value was calculated [42]. A high GP value (≈+0.4) is characteristic for a rigid lipid bilayer with densely packed lipid molecules, i.e., the membrane gel state, whereas a low GP value (≈−0.2) is characteristic for less densely packed lipid bilayers, i.e., the fluid membrane state. As these values are largely independent of the lipid head groups and acyl chains, changes in the GP value can provide information about changes in the lipid order upon the addition of substances [43].

**Figure 3:**
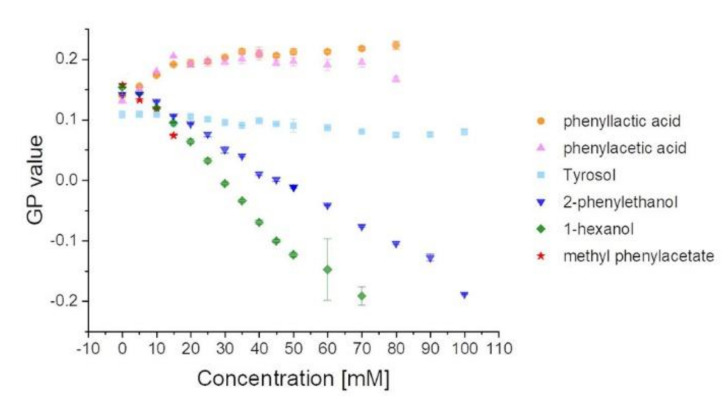
2-PEtOH and derivatives affect the structure of the model membranes. GP values determined at increasing substance concentrations are shown. The values indicate a fluidizing effect for the more hydrophobic substances 1-hexanol, 2-PEtOH, and methyl phenylacetate. Tyrosol seems to be largely ineffective, while phenyllactic acid and phenylacetic acid seem to have a slight ordering effect.

To this end, unilamellar liposomes were prepared from *E. coli* lipids containing 2 µM Laurdan as well as increasing concentrations of the substances analyzed here. Changes in lipid packing were determined via laurdan fluorescence spectroscopy and illustrated as changes in the GP values (Figure 3). For further details, see Materials and Methods. 

2-PEtOH and 1-hexanol are well known to increase membrane fluidity [1,2,8]. In line with this, the GP values measured here with increasing 2-PEtOH and 1-hexanol concentrations, respectively, are constantly decreasing (Figure 3), indicating a membrane fluidizing effect of all substances. For the maximal tested concentrations of the two substances, 70 mM for 1-hexanol and 100 mM for 2-PEtOH, the GP value is around −0.2. This value is characteristic of a bilayer in the fluid (liquid crystalline) phase [42,43]. Here, 2-PEtOH acts like 1-hexanol, although the impact of 1-hexanol on the lipid acyl chain order was more pronounced already at lower concentrations. 

Yet, even though Tyrosol binds solely to membrane surfaces, an impact on the lipid order might be expected. However, we did not see any change of the GP values with increasing Tyrosol concentrations, and thus, apparently, surface adhesion of Tyrosol does not (significantly) affect the membrane structure.

Further, for phenylacetic acid and phenyllactic acid, it was suggested that they could incorporate into membranes [14,17,20,24], which is in line with the calculated logP values (Table 1). Yet, in contrast to 2-PEtOH, the addition of both phenyllactic acid as well as phenylacetic acid to *E. coli* lipid membranes did result in increasing GP values, which remained constant at concentrations larger than 15 mM. Thus, phenylacetic acid and phenyllactic acid both appeared to increase rather than decrease the membrane lipid order creating a more rigid membrane. Nevertheless, the membrane ordering effect was much lower than the disordering effect of 2-PEtOH or 1-hexanol.

To estimate whether the reverse impact of the two acids, compared to 2-PEtOH, might be caused by the negative charge, we additionally analyzed the impact of the methyl ester of phenylacetic acid, methyl phenylacetate, on the membrane structure. The addition of methyl phenylacetate to the model membranes resulted in decreasing GP values, as observed with 2-PEtOH or 1-hexanol. Consequently, membrane incorporation of methyl phenylacetate increased the membrane fluidity, and thus masking the negative charge of phenylacetic acid seemed to have a significant impact on the membrane activity (as further discussed below).

### 3.2. 2-PEtOH and Derivatives Are Bacteriostatic

**Figure 4:**
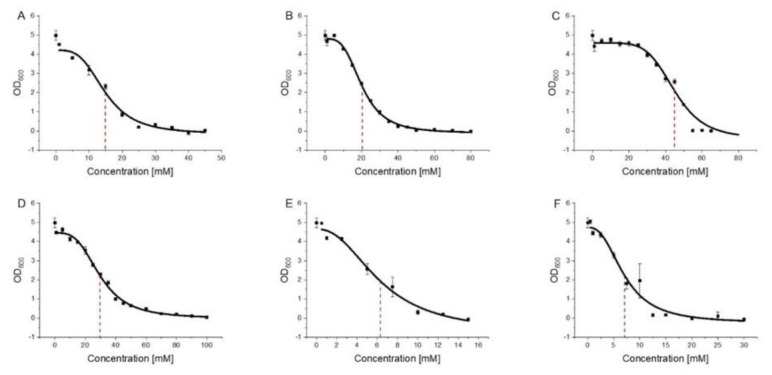
Determination of MIC50 values. The OD_600_ was plotted against the substance concentration and a dose-response fit was performed for (**A**) 2-PEtOH, (**B**) phenylacetic acid, (**C**) phenyllactic acid, (**D**) Tyrosol, (**E**) methyl phenylacetate, and (**F**) 1-hexanol (n = 3, ±SD). The corresponding MIC50 values are given in Table 1.

For all substances, except for Tyrosol, we showed that membrane binding had an impact on the membrane structure. Next, to test whether this membrane activity correlates with a potential bacteriostatic activity of the substances, the impact of 2-PEtOH, phenylacetic acid, phenyllactic acid, methyl phenylacetate, Tyrosol, and 1-hexanol on the growth of the bacterium *E. coli* was tested. For 2-PEtOH, it was already shown that it decreases bacterial growth starting at concentrations as low as 8 mM with explicit effects at 12–16 mM [7]. We followed bacterial growth in presence of increasing substance concentrations to calculate the (non-lethal) amount of substance that inhibits 50% bacterial growth (Figure 4). 

This minimal inhibitory (MIC50) value is a measure of the antimicrobial activity of compounds [44].

In excellent agreement with literature values, we here observed a clear effect of 2-PEtOH on *E. coli* growth with an MIC50 value of ~15 mM [5,6,7,8,9]. As for phenylacetic acid, we likewise observed a bacteriostatic effect with an MIC50 value of ~20 mM. However, when we masked the negative charge and analyzed the methyl phenylacetate instead, the MIC50 value was lowered to ~6.3 mM, a value lower than 2-PEtOH and in the same range as observed with 1-hexanol. This showed that masking the negative charge not only considerably affected the membrane activity of (methyl) phenylacetate (Figure 3) but also significantly enhanced its bacteriostatic efficiency. Surprisingly, while Tyrosol did not integrate into and affect the structure of biomembranes but rather lies flat on membrane surfaces (Figure 2H), it still affected the *E. coli* growth with an MIC50 value of ~30 mM [16,40]. The naturally produced 2-PEtOH derivative phenyllactic acid was least active with an MIC50 as high as ~45 mM. The determined MIC50 values are summarized in Table 1 for each of the tested substances.

### 3.3. Hydrophobicity, Membrane Fluidity, and Bacterial Growth Correlate

The here-analyzed molecules with the highest logP values decreased the membrane order with increasing concentration, whereas phenylacetic acid and phenyllactic acid, which both have a low logP value, showed increasing GP values with increasing concentration. Thus, the higher the overall hydrophobicity of a molecule, the higher its fluidizing impact on a membrane. Nevertheless, as all molecules, except the control 1-hexanol as well as Tyrosol, had a methylene benzene group, the chemistry of the substituents was evidently important for the membrane activity of 2-PEtOH derivatives. In contrast to 2-PEtOH, phenylacetic acid and phenyllactic acid did not decrease but slightly increased the order of a lipid bilayer. Yet, when masking the negative charge via the formation of a methyl ester, methyl phenylacetate showed an even increased membrane fluidizing activity compared to 2-PEtOH. Thus, the incorporation of the hydrophobic methylene benzene group into the hydrophobic membrane core region, as well as (polar) interactions within the lipid head group region, likely together affect the membrane activity of 2-PEtOH derivatives (as well as of other substances). This appears to be nicely reflected by the calculated overall hydrophobicity, i.e., the calculated logP values.

**Figure 5:**
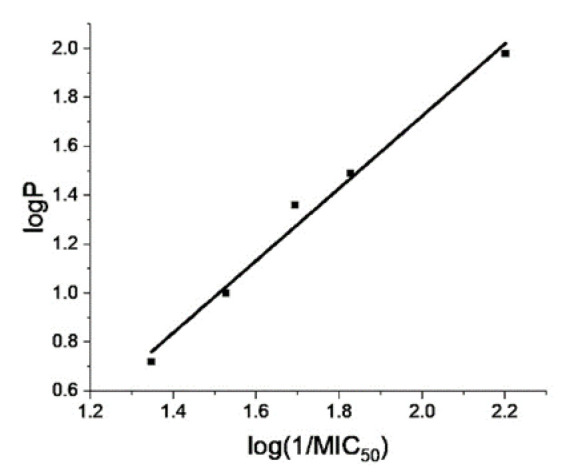
The logP values of 2-PEtOH linearly correlate with the log(1/MIC_50_) values. The logP values of 2-PEtOH and derivatives is plotted against the respective log(1/MIC_50_) values. The correlation coefficient is r^2^ = 0.987.

Based on several subsequent studies, the bacteriostatic activity of 2-PEtOH was linked to biological membranes, albeit the exact mode of action is still unclear, and other target structures were also discussed [4,7,8,9]. Yet, the calculated logP values nicely correlated with the determined MIC50 values (Figure 5), and with an increasing logP value, the MIC50 decreased. In fact, when we plot the logarithm of the MIC50 values determined for the 2-PEtOH derivatives against the calculated logP values, we obtained a linear correlation with an r^2^ value of 0.987 (Figure 5). Thus, the hydrophobicity of the molecules, i.e., their calculated propensity to partition into biomembranes, correlated with the bacteriostatic activity, which indicated that the partition coefficient significantly determined the biological activity of the substances and links the bacterotoxic activity of the substances to biomembranes. 

In most cases, a clear effect on membrane lipid order was already observed in the liposome-based assay at the MIC50 values, and, thus, changes in general membrane properties could well have an important impact on the bacteriostatic activity of the 2-PEtOH derivatives. Yet, there was no general correlation between the bacteriostaticity and the observed effect on the membrane structure: All substances clearly had an impact on bacterial growth, yet some substances increased the lipid order (phenylacetic acid, phenyllactic acid), Tyrosol did essentially not affect the membrane structure, whereas the remaining substances decreased the lipid order. Furthermore, it has to be noted that all molecules analyzed here might also have additional cellular targets. 

In summary, our results indicate a correlation between the hydrophobicity of the 2-PEtOH derivatives analyzed here and their respective bacteriostatic activity, and our results link the biological activity of the molecules to cellular membranes.

## Figures and Tables

**Table 1:** LogP and MIC50 values of 2-PEtOH and derivatives.

Substance	logP	MIC_50_
phenyllactic acid	0.72	44.97
Tyrosol	1.00	29.74
phenylacetic acid	1.36	20.28
2-phenylethanol	1.49	14.89
methyl phenylacetate	1.98	6.30
1-hexanol	2.13	7.05

The logarithm of the partition coefficient (logP) values of the substances, which provide information as to membrane partitioning of a molecule, were calculated with the online Molinspiration software v2016.10. The higher the value, the more hydrophobic the molecule and the higher will be the fraction of the membrane incorporated substance. The minimal inhibitory (MIC50) value provides information as to the bacteriostatic potential of a molecule (compare Figure 4). The higher this value, the less bacteriostatic a substance.

## Data Availability

All data is contained within this article.
